# Static Regulation and Dynamic Evolution of Single‐Atom Catalysts in Thermal Catalytic Reactions

**DOI:** 10.1002/advs.201801471

**Published:** 2018-11-27

**Authors:** Hongliang Li, Menglin Wang, Laihao Luo, Jie Zeng

**Affiliations:** ^1^ Hefei National Laboratory for Physical Sciences at the Microscale Key Laboratory of Strongly‐Coupled Quantum Matter Physics of Chinese Academy of Sciences National Synchrotron Radiation Laboratory Department of Chemical Physics University of Science and Technology of China Hefei Anhui 230026 P. R. China

**Keywords:** dynamic evolution, single‐atom catalysts, static regulation, thermal catalytic reactions

## Abstract

Single‐atom catalysts provide an ideal platform to bridge the gap between homogenous and heterogeneous catalysts. Here, the recent progress in this field is reported from the perspectives of static regulation and dynamic evolution. The syntheses and characterizations of single‐atom catalysts are briefly discussed as a prerequisite for catalytic investigation. From the perspective of static regulation, the metal–support interaction is illustrated in how the supports alter the electronic properties of single atoms and how the single atoms activate the inert atoms in supports. The synergy between single atoms is highlighted. Besides these static views, the surface reconstruction, such as displacement and aggregation of single atoms in catalytic conditions, is summarized. Finally, the current technical challenges and mechanistic debates in single‐atom heterogeneous catalysts are discussed.

## 1 Introduction

More than 80% of chemical reactions are related to catalytic processes.^1^ Typical catalysts involve homogenous and heterogeneous catalysts. Homogeneous catalysts usually exhibit higher activity and selectivity compared with heterogeneous counterparts. Moreover, the uniform active sites and the controllable coordination environment in homogeneous catalysts enable deep understanding of catalytic mechanisms. However, homogeneous catalysts generally suffer from the poor stability and complex separation procedure. Although heterogeneous catalysts are able to avoid these disadvantages, the lower atomic utilization efficiency and ill‐defined active sites relative to homogenous ones bring about both economic and academic concerns. To this end, single‐atom catalysts have aroused wide interests from researchers, promisingly bridging the gap between homogenous and heterogeneous catalysts.^2^

Single‐atom catalysts are composed of isolated metal atoms/ions which are dispersed on the surface of supports such as metal oxides, carbides, sulfides, zeolites, and graphene.^3^ Besides maximizing the utilization efficiency of metal atom, single‐atom catalysts greatly enhanced the catalytic activity and selectivity because of their distinct coordination structures.^4^ Specifically, the quantum size effect leads to the significant electron confinement, resulting in a discrete energy level distribution and a distinctive gap between the highest occupied molecular orbital and the lowest unoccupied molecular orbital. The unsaturated coordination of metal atoms enables these sites to adsorb and react with substrate molecules. The strong metal–support interaction guarantees electron transfer between metal atoms and supports. In addition, heteroatom effect gives rise to asymmetrical spin and charge density.

In the recent years, single‐atom catalysts have been widely applied in reactions driven by thermal, electric, and light energy.^5^ The applications of single‐atom catalysts are briefly summarized in **Table** **1**. They exhibited catalytic performance different from their nanocrystal counterparts. For instance, CO oxidation over metallic Pt nanocrystals follows a Langmuir–Hinshelwood mechanism with competitive adsorption between CO and O_2_ at low temperatures.^6^ As for atomically dispersed Pt on CeO_2_, the reaction follows a Mars–van Krevelen mechanism with the dissociative O_2_ adsorption and activation on CeO_2_, thereby circumventing the competitive adsorption between O_2_ and CO on Pt.^7^ Another typical example involves the selective hydrogenation of 1,3‐butadiene, where Pt single atoms achieved high selectivity for butenes due to the absence of continuous Pt ensembles for hydrocarbon decomposition.[[qv: 8a]] Moreover, Pd single atoms on exfoliated graphitic carbon nitride even outperformed homogeneous systems toward Suzuki coupling.[[qv: 8b]]

**Table 1:** Applications of single‐atom catalysts

Catalysts	Applications	References
Pt_1_/FeO*_x_*, Pt_1_/Fe_3_O_4_, Au_1_/Def‐TiO_2_, Au_1_/CuO, Pt_1_/CeO_2_, Pd_1_Au_24_, Pt_1_/θ‐Al_2_O_3_, 0.5 wt% Rh‐ZSM‐5, Rh_1_/ZrO_2_	Oxidation of CO, alcohol, NO, methane	[[qv: 2c,e,3a,e,11c,13a,b,26d,42]]
0.5% Fe©SiO_2_	Nonoxidative conversion of methane	33
Pt_1_/α‐MoC, Rh_1_/VO_2_	Hydrogen production from methanol, ammonia borane	[[qv: 12a,27]]
Pt_1_/MoS_2_, Pt_1_Ni, Pd_1_/graphene, Pd_1_/TiO_2_, Pd_1_/Cu, Pt_1_/Cu, PdIn/MgAl_2_O_4_	Selective hydrogenation of CO_2_, nitro compounds, butadiene, styrene, acetylene, aldehydes	[[qv: 5b,8a,11a,17b, 20,21a,30]]
Pd_1_/exfoliated graphitic carbon nitride	Cross‐coupling reactions	[[qv: 8b]]
Au—O(OH)*_x_*, Ir_1_/FeO*_x_*	Water‐gas shift	[[qv: 13e,28]]
Ag_1_Cu, Pt_1_/ZnO, Au_1_/ZnO, Rh_1_/*x*Sm_2_O_3_–yCeO_2_–Al_2_O_3_	Steam reforming, methanol reforming	[[qv: 3d,11b,13f]]
Rh_1_/CoO, Rh_1_/ZnO, Pt_1_/Al_2_O_3_, Au_1_/C, Co_1_/MoS_2_	Hydroformylation, hydrosilylation, hydrochlorination, hydrodeoxygenation	[[qv: 3f,14a,16b,25,38]]
Ru_1_/N—C, Co_1_—NC, Pt_1_—TiN, C—N—Co	Electrochemical reduction of CO_2_, N_2_, O_2_	[[qv: 5e,g,12c,15e]]
Pt_1_/MoS_2_, HCl‐Ni@C, NiN_4_C_4_, Co‐C_3_N_4_/CNT	Electrochemical evolution of H_2_, O_2_	[[qv: 3c,14b,15f,g]]
Ni_1_N‐graphene, Pd_1_/g‐C_3_N_4_, Pt_1_/g‐C_3_N_4_, Pt_1_—CN, Al–TCPP–0.1Pt	CO_2_ photoreduction, photocatalytic H_2_ evolution	[[qv: 2b,5f,10b,15h]]

Herein, we report the recent progresses in single‐atom catalysis toward thermal catalytic reactions from the perspectives of static regulation and dynamic evolution. As a prerequisite for catalytic investigation, we briefly review the approaches to fabricating and characterizing single‐atom materials. For static regulation, we discuss the mutual metal–support interaction from the views of both metal single atoms and supports, and highlight the synergetic interaction in single‐atom catalysis. As for dynamic evolution, we also summarize the surface reconstructions such as displacement and aggregation of single atoms in operando conditions. In the last part, we reemphasize the recent breakthrough in heterogeneous catalysis and prospect the development of this field.

## 2 Synthetic Approaches and Characterization Methods

### 2.1 Synthetic Approaches

**Figure 1:**
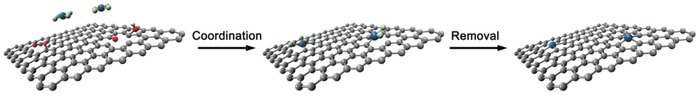
Illustration of preparing single‐atom catalysts. Grey, red, blue, and green spheres represent the atoms in supports, the anchoring sites, the target metal atoms, and the ligands.

Fabrication of single‐atom catalysts is the prerequisite for further investigation of their catalytic performance and mechanisms. The extremely high surface energy of isolated metal atoms makes it challenging to prevent them from aggregation under harsh preparation or catalytic conditions. To this end, great efforts have been devoted to physically or chemically confining and stabilizing isolated metal atoms from aggregating into clusters or nanoparticles. Recently, there are several reviews on the synthesis of single‐atom catalysts.^9^ In this work, we briefly discuss these approaches. Typical synthetic approaches involve two steps: coordinating the ensemble composed of active metal species with the supports; removing the residual ligands from single metal sites (**Figure** **1**).

The coordination step requires the deliberate selection of supports and the delicate synthetic operation. Supports afford the physical isolation or chemical stabilization of metal single atoms. Typical supports for single‐atom catalysts include microporous matrices, metal‐containing supports, and metal‐free supports. Microporous matrices such as zeolites, metal–organic frameworks, and covalent–organic frameworks offer micropores to physically confine or chemically graft metal single atoms.^10^ Metal‐containing supports involve metal nanocrystals, metal carbides, metal oxides, metal sulfides, and so on. Metal surfaces such as Cu, Ni, and Au anchor single atoms via strong metal–metal bonds.[[qv: 4e,11]] Such interaction also enable the stabilization of single atoms on metal‐like materials such as α‐MoC, WC*_x_*, and TiC.^12^ Reducible metal oxides (e.g., TiO_2_, CeO_2_, FeO_x_, CoO*_x_*, and WO*_x_*) stabilize single atoms through defects such as oxygen vacancies.[[qv: 3a,13]] Single atoms can also be embedded in metal sulfides such as MoS_2_ via doping.^14^ When metal‐free materials such as graphene, g‐C_3_N_4_, BN, and HSC serve as the supports, chemical bonds are formed between single metal atoms and their coordinating atoms (e.g., C, O, N, and S).^15^ In addition, synthetic experiments also need to be delicately operated. During wet‐chemistry methods, one can achieve the deposition of single atoms via either decreasing the amount of metal loading or controlling the precursor reduction at a proper rate to prevent self‐nucleation.^16^ Another approach which can precisely control the synthesis of single‐atom catalysts is atomic layer deposition which relies on sequential self‐terminating reactions between a solid surface and gas phase precursor molecules.^17^ More importantly, the experimental operation needs to adapt for the selection of supports. Datye and co‐workers trapped Pt single atoms on CeO_2_ at high temperature. This approach requires a supply of mobile atoms and a support that can bind the mobile species.^18^

The residual ligands can saturate the coordination of metal single atoms to decrease their catalytic activity, or destabilize the metal atoms to trigger aggregation. A typical method to remove ligands is the combustion under O_2_ atmosphere.[[qv: 17a,19]] In addition to such harsh treatment, Zheng and co‐workers reported the removal of Cl^−^ ligands on Pd single atoms under mild conditions via a photochemical route.^20^ Once Pd atoms coordinated with Cl^−^ ligands on TiO_2_ were exposed to ultraviolet (UV) irradiation, electron–hole pairs were generated on TiO_2_ nanosheets. Electrons were trapped in Ti‐3d orbitals to form Ti^3+^ sites, while holes broke Ti—O bonds between glycolate and TiO_2_, leading to the formation of ethylene glycolate (EG) radicals. The UV‐generated EG radicals promoted the removal of Cl^−^ on Pd and the stabilization of individual Pd atoms via Pd—O bonds.

### 2.2 Characterization Methods

**Figure 2:**
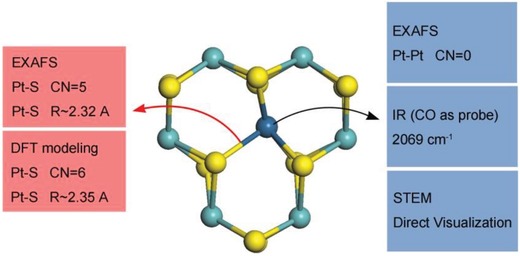
Characterization of Pt single atoms in MoS_2_ via different techniques. Reproduced with permission.^30^ Copyright 2018, Nature Publishing Group.

To fully convince the fabrication of single‐atom catalysts, we need to not only directly visualize the single atoms, but also guarantee the absence of clusters or nanoparticles. Accordingly, the characterization of single‐atom catalysis requires a series of complementary techniques to prove the exclusive existence of single atoms. These techniques include atomic resolution aberration‐corrected scanning transmission electron microscopy (STEM), X‐ray absorption fine structure (XAFS), infrared (IR) spectroscopy, and theoretical calculations (**Figure** **2**).

Atomic resolution aberration‐STEM has been utilized as a powerful technique for the direct visualization of atomically dispersed metal atoms on the supports. The contrast in the HAADF‐STEM image is associated with the atomic number of the observed atom. When metal atoms are singly dispersed in the detected region, the spots with sharp contrast are separated far apart instead of aggregating into patches.^21^ However, the observation of single atoms in STEM images is a only necessary but not sufficient condition for identifying atomically dispersed catalysts. Specifically, the STEM image only offers information about a partial region of a catalyst, but is unable to ensure the adaptability of this local area to the overall structure of each synthesized catalyst. Moreover, the STEM does not apply to distinguishing metal atoms (e.g., Cu and Zn atoms) with similar atomic numbers.

As a typical complement to STEM, XAFS afford the information about the statistical average of the overall structure. XAFS spectrum generally involves X‐ray absorption near‐edge spectroscopy (XANES) and extended XAFS (EXAFS). XANES reflects the oxidation state and symmetry (e.g., octahedral coordination) of the absorbing atom.^22^ EXAFS offers the species of atoms coordinated with the absorbing one, the coordination number, and the bond distance.^22^ For a single‐atom catalyst, only the coordination with foreign atoms was detected in the absence of the bonding with the same metal atoms.^21^

Another technique to identify single‐atom catalysts is IR with the help of probe molecules. By detecting the vibrational intensity and frequency of the probe molecules, we obtain the oxidation states and coordination environment of the active centers. For example, we utilize CO molecules to distinguish Pt single atoms and Pt clusters.^23^ The typical IR characteristics of Pt single atoms are listed. First, the stretching frequency (2080–2170 cm^−1^) for CO on single Pt^δ+^ atoms is shifted to 40–50 cm^−1^ higher than that (2030–2100 cm^−1^) for CO linearly bonded on Pt^0^ in clusters. Second, the peak (1750–1950 cm^−1^) for bridge‐bonded CO on two Pt atoms in clusters is absence for Pt single atoms. Moreover, the stretching frequency for CO on isolated Pt atoms is independent on the coverage of CO due to spatial separation of Pt atoms. In single‐atom catalysts, there are no adjacent CO molecules in close enough proximity to induce dipole–dipole coupling responsible for frequency shifts. As for CO adsorbed on Pt clusters, the stretching frequency redshifts with decreasing the coverage of CO due to dipole–dipole coupling. Adjacent CO molecules vibrate in unison, giving rise to a lower vibrational energy at lower coverage.

Besides the experimental techniques, theoretical calculations also play a pivotal role in determining the specific configuration of the active sites. Compared with metal clusters or nanoparticles, single‐atom catalysts offer an ideal platform which is significantly simplified in theoretical calculations. The detailed information from microscopic and EXAFS data makes it convenient and convincing to establish the theoretical model. For example, Prof. Tsang applied the calculations based on density functional theory (DFT) to obtain the model of Co single atoms on Mo atop sites in MoS_2_.[[qv: 14a]] With the help of DFT, Li and co‐workers found that the Au atom was twofold coordinated by Ti atoms in the defective TiO_2_ nanosheets.[[qv: 13a]]

## 3 Static Regulation of Single‐Atom Catalysts in Thermal Catalytic Reactions

### 3.1 Metal–Support Interaction

Metal–support interaction not only plays a pivotal role in anchoring metal single atoms on the supports, but also makes a remarkable impact on catalytic performance.^24^ Enhanced coordination of metal single atoms with a support varies the electronic properties of catalysts. In contrast, weak metal interaction suppresses catalytic processes that occur on multiatom sites. Optimizing the catalytic performance of single‐atom catalysts requires proper metal–support interaction via deliberate selection of an appropriate support. For instance, Ma and co‐workers demonstrated that the interaction between Pt single atoms and α‐MoC enabled effective methanol‐reforming reaction because of abundant surface hydroxyls produced on α‐MoC.[[qv: 12a]] Hutchings and co‐workers found that the different Au‐Cl coordination in Au single‐atom catalysts induced the varied ratio of Au(I):Au(III), resulting in different activity toward the production of vinyl chloride monomer.^25^

**Figure 3:**
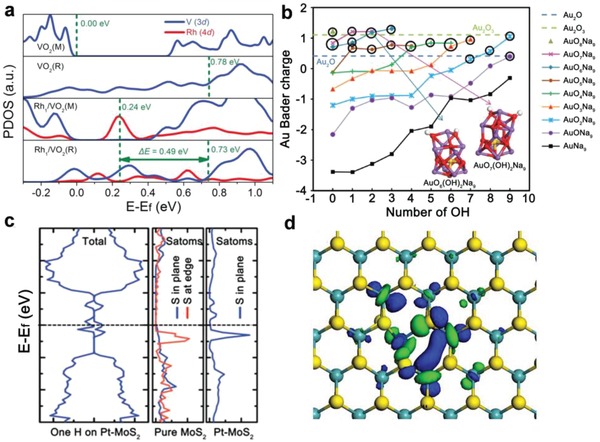
a) Calculated projected densities of states (PDOS) of VO_2_(M), VO_2_(R), Rh_1_/VO_2_(M), and Rh_1_/VO_2_(R). Reproduced with permission.^26^ Copyright 2017, Wiley‐VCH. b) Bader charge of Au in AuO_x_(OH)_y_Na_9_ cluster sites. Reproduced with permission.^27^ Copyright 2014, American Association for the Advancement of Science. c) Total DOS for one H adsorbed on Pt–MoS_2_, and PDOS of in‐plane and edge S atoms from pure MoS_2_ and Pt–MoS_2_. Reproduced with permission.^28^ Copyright 2015, Elsevier. d) The profile distributions of the LUMO of Pt_1_/MoS_2_, respectively. Reproduced with permission.^30^ Copyright 2018, Nature Publishing Group.

From the perspective of metal single atoms, their electronic properties such as the highest occupied state (HOS) and charge are altered by the supports which vary in band structures, coordination number, etc., to bind single atoms.^26^, ^27^, ^28^ For example, Wang et al. quantitatively depicted the profile of metal–support interaction for single‐atom catalysts from the perspective of the HOS. They dispersed Rh single atoms on the surface of VO_2_ (Rh_1_/VO_2_).^27^ During NH_3_BH_3_ hydrolysis over Rh_1_/VO_2_, the activation energy decreased by 38.7 kJ mol^−1^ after the metal–insulator transition of supports from monoclinic VO_2_(M) to rutile VO_2_(R). The kinetic analysis indicated that the activation of proton served as the rate‐limiting step. Based on the first‐principle calculations, the doping of Rh in VO_2_(M) arouses new occupied states in the band gap of VO_2_(M), whereas the HOS of Rh_1_/VO_2_(R) is at the energy comparable to the Fermi level of VO_2_(R) (**Figure** **3**a). The divergence in the HOS between Rh_1_/VO_2_(M) and Rh_1_/VO_2_(R) was 0.49 eV (47.3 kJ mol^−1^), which was close to that (38.7 kJ mol^−1^) of activation energy. In this regard, the researchers associated the difference of apparent activation energy between two phases of VO_2_ with the HOSs of Rh single atoms. In addition, Flytzani–Stephanopoulos and co‐workers reported that the Bader charge of Au in AuO*_x_*(OH)*_y_*Na_9_ was tuned through varying the number of electron‐withdrawing groups (O/OH) on zeolites (Figure 3b).^28^

From the perspective of supports, the initially inert atoms in the supports can also be activated by their coordinated metal single atoms. Bao and co‐workers reported that Pt single atoms triggered the activity of in‐plane S atoms of MoS_2_ toward hydrogen evolution reaction.[[qv: 14b]] Based on DFT calculations, the introduction of Pt single atoms increased the electronic states of in‐plane S sites below the Fermi level (Figure 3c). The activated in‐plane S sites exhibited comparable electronic states to that of the edge S atoms that generally served as the active site for hydrogen evolution as a widespread consensus.^29^ Similar phenomenon was also observed by Li et al. in the study of neighboring Pt monomers on MoS_2_ (Pt_1_/MoS_2_) toward CO_2_ hydrogenation.^30^ The molecular orbital analysis indicated the electron transfer between the Pt atom and its bonded S atoms (Figure 3d). Pt atoms activated their vicinal S atoms to dissociate H_2_ and adsorb intermediates. In neighboring Pt monomers, a part of the activated S atoms are shared or adjacent. Those S atoms bridged the range of influence exerted by two Pt atoms and thereby reflected the synergetic interaction.

### 3.2 Synergetic Interaction

To avoid the aggregation of single atoms, the mass loading is generally controlled relatively low. The low loading leads to the separation of single atoms far apart, resulting in the negligible interaction between single atoms. However, shortening the distance between two single atoms arouses distinct catalytic performance. Goodman and co‐workers found that a properly spaced pair of noncontiguous Pd sites on Au(111) surface enabled the coupling between a surface ethylenic and acetate species, and thereby exhibited higher activity for vinyl acetate formation than isolated Pd sites.^31^ In addition, Yardimci et al. reported that Rh dimers in [Rh_2_(C_2_H_5_)_2_] species promoted the scission of H—H bond, resulting in more efficient hydrogenation of ethylene than Rh monomers in [Rh(C_2_H_4_)_2_] complexes.[[qv: 22a,32]] Bao and co‐workers revealed that single Fe sites embedded in a silica matrix enabled direct, nonoxidative conversion of methane, whereas adjacent Fe sites led to C—C coupling, further oligomerization and coke deposition.^33^

**Figure 4:**
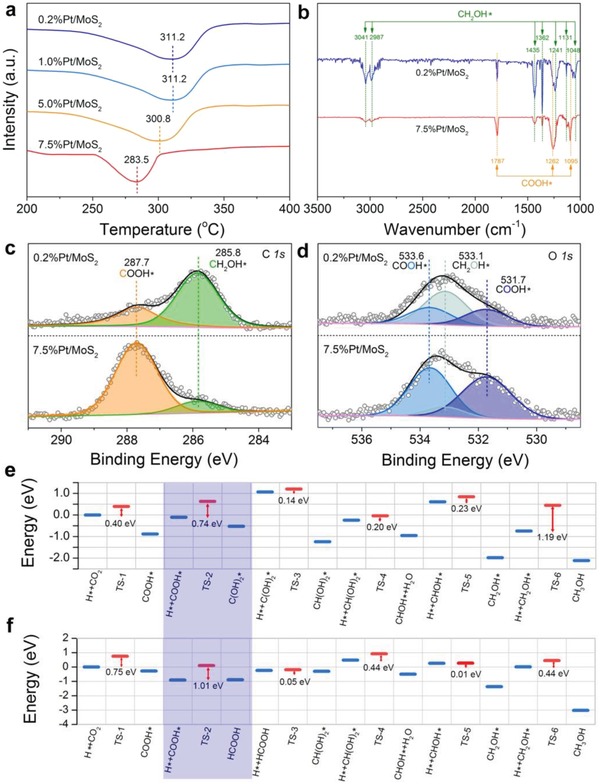
a) H_2_‐TPD profiles of atomically dispersed Pt/MoS_2_ with different Pt loadings. b) In situ DRIFT spectra of 0.2%Pt/MoS_2_ and 7.5%Pt/MoS_2_. c,d) In situ XPS spectra of C_1S_ and O_1S_ for 0.2%Pt/MoS_2_ and 7.5%Pt/MoS_2_. e,f) Optimized reaction paths in CO_2_ hydrogenation for isolated and neighboring Pt monomers on MoS_2_, respectively. Reproduced with permission.^30^ Copyright 2018, Nature Publishing Group.

Recently, Zeng and co‐workers revealed the synergetic interaction between Pt monomers by facilely increasing the Pt mass loading up to 7.5% while still maintaining the atomic dispersion of Pt. In Pt_1_/MoS_2_,^30^ Pt atoms replaced Mo atoms in MoS_2_ nanosheets, wherein every Pt atom and its directly bonded S atoms composed an “active center.” When two active centers were partly overlapped or adjacent, the two relevant Pt atoms were regarded as neighboring monomers. During CO_2_ hydrogenation, neighboring Pt monomers exhibited higher activity than isolated ones. The researchers further investigated the catalytic mechanism of different types of Pt monomers by combining temperature‐programmed desorption (TPD), in situ diffuse reflectance infrared Fourier transform (DRIFT), in situ X‐ray photoelectron spectroscopy (XPS), and DFT calculations. They found neighboring Pt monomers promoted the dissociation of H_2_ relative to isolated ones (**Figure** **4**a). Moreover, CO_2_ was converted into methanol without experiencing the formation of formic acid intermediates over isolated Pt monomers (Figure 4b–e). In contrast, neighboring Pt monomers worked in synergy to alter reaction pathways, where CO_2_ undergoes sequential transformation into formic acid and methanol (Figure 4b–f).

## 4 Dynamic Evolution of Single‐Atom Catalysts in Thermal Catalytic Reactions

During catalytic reactions such as CO_2_ hydrogenation, Fischer–Tropsch synthesis (FTS) and CO oxidation, the surface of catalysts is unable to retain its original structures due to the corrosion of acidic/alkaline environment, the adsorption of substrate molecules, the coordination of solvent molecules, or other factors.^34^ For instance, during FTS, Fe‐based catalysts tend to form iron‐carbide phases such as cementite, Hägg carbides, and hexagonal carbides, owing to the high affinity of Fe atoms to C atoms cleaved from CO.^35^ In addition, the surface carbonization has been reported to account for the deactivation of Co‐based catalysts during FTS.^36^ Although single atoms are anchored on the support surface via strong metal–support interaction, the high surface energy of single atoms usually results in the surface reconstruction such as displacement and aggregation in catalytic reactions.^37^, ^38^, ^39^, ^40^, ^41^, ^42^, ^43^

**Figure 5:**
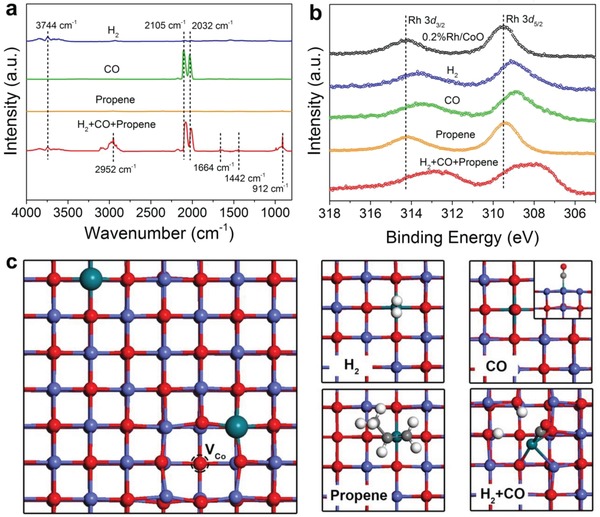
a) In situ DRIFT spectra of 0.2%Rh/CoO after the treatment of the sample with different gas at 100 °C. b) In situ XPS spectra of 0.2%Rh/CoO before and after the treatment of the sample with different gases at 100 °C. c) The model of Rh_1_/CoO. Two Rh atoms occupy the position of two Co atoms, together with the involvement of a Co vacancy. Top views of the adsorption configurations of H_2_, CO, and propene on Rh_1_/CoO, respectively. Reproduced with permission.^38^ Copyright 2016, Nature Publishing Group.

Wang et al. reported that the adsorption of CO and H_2_ induced the displacement of Rh single atoms on CoO (Rh_1_/CoO) surface during hydroformylation reaction.^38^ During the hydroformylation of propene, Rh_1_/CoO achieved the TOF number of 2065 h^−1^ and selectivity of 94.4% for butyraldehyde. They found that sole propene was weakly adsorbed on Rh_1_/CoO, whereas the adsorption of propene was prominently facilitated in the atmosphere containing both H_2_ and CO (**Figure** **5**a,b). Based on the theoretical calculation, the position of Rh single atoms did not change during the adsorption of sole CO, H_2_, or propene (Figure 5c). Interestingly, Rh single atoms deviated from the lattice point after the adsorption of both H_2_ and CO on Rh_1_/CoO (Figure 5c). The displacement of Rh atoms promoted the adsorption of propene, as the adsorption energy of propene significantly increased by 0.34 eV. As such, Rh single atoms on CoO underwent displacement from their original positions during hydroformylation reaction, facilitating the adsorption and activation of reactants.

Reactant molecules can also induce mobility of metal single atoms and lead to agglomeration into clusters. Rousseau and co‐workers theoretically predicted that CO adsorption gave rise to the reconstruction of Au nanoparticles into low coordinated and mobile Au—CO species.^39^ This prediction was later confirmed by room‐temperature STM experiments.^40^ Further researches revealed that the Au single atoms linked with O atoms existed only in operando CO oxidation and returned to Au nanoparticle after the completion of reaction.^41^ Parkinson and co‐workers demonstrated that the strong interaction between CO and Pt adatoms led to the formation of Pt carbonyls (Pt_1_—CO) and weakened the Pt—O bonds.^42^ Interestingly, Pt_1_—CO monomers aggregated into clusters composed of different Pt atoms under different CO coverage. Based on in situ TEM, Corma and co‐workers directly visualized the dynamic reversible transformation between atomically dispersed Pt species and clusters/nanoparticles during CO oxidation at different temperatures.^43^

## 5 Conclusions and Prospects

Single‐atom catalysts behave similar to homogeneous catalysts and retain heterogeneous catalysts' advantage of recycling, thereby bridging the huge gap between these two catalyst systems. Recent years have witnessed the great progresses in the synthesis and characterization of single‐atom catalysts. Moreover, the recent researches greatly advances the understanding of how the single atoms and supports interact mutually, whether two single atoms work individually or cooperate in synergy, and how the single atoms dynamically evolve during catalytic reaction. However, there still remain various technical challenges and mechanistic debates in single‐atom catalysts.

Advanced characterization technologies are always necessary for the investigation of single‐atom catalysis. Direct visualization of different metal atoms (e.g., Cu and Zn atoms) with similar atomic numbers still remains as a challenge. Besides, in situ techniques are required to bridge the pressure gap for better understanding the catalytic process such as the rate‐limiting step, the adsorbed intermediates, and the evolution of single atoms.

Precise control over the number of active metal atoms represents a pivotal prospective of the development of single‐atom catalysis. A lot of reactions such as C—C coupling, A^3^ coupling, esterification, etc., require more than one active site. More atoms mean more geometrical patterns, bringing larger challenges in both synthesis and mechanistic studies. Establishing the database of the active ensemble containing different number of atoms is of vital importance to heterogeneous catalysis.

More attention can be turned to the atoms or ligands coordinated with the metal single atoms. Utilizing metal single atoms to trigger the activity of inert atoms in supports will largely extends the application of single‐atom catalysis. Moreover, the study of this field also helps to determine the real active sites during catalytic reactions and understand the mechanism in a more comprehensive view.

## Conflict of Interest

The authors declare no conflict of interest.
